# Can We Systematically Review Studies That Evaluate Complex Interventions?

**DOI:** 10.1371/journal.pmed.1000086

**Published:** 2009-08-11

**Authors:** Sasha Shepperd, Simon Lewin, Sharon Straus, Mike Clarke, Martin P. Eccles, Ray Fitzpatrick, Geoff Wong, Aziz Sheikh

**Affiliations:** 1 Department of Public Health, University of Oxford, Oxford, United Kingdom; 2 Norwegian Knowledge Centre for the Health Services, Oslo, Norway; 3 Health Systems Research Unit, Medical Research Council of South Africa, South Africa; 4 Department of Medicine, University of Toronto, Toronto, Ontario, Canada; 5 UK Cochrane Centre, Oxford, United Kingdom; 6 School of Nursing and Midwifery, Trinity College Dublin, Ireland National Institute for Health Research, Oxford, United Kingdom; 7 Institute of Health and Society, Newcastle University, Newcastle Upon Tyne, United Kingdom; 8 Research Department of Primary Care and Population Health, UCL, London, United Kingdom; 9 Centre for Population Health Sciences, University of Edinburgh, Edinburgh, United Kingdom; 10 CAPHRI, University of Maastricht, Maastricht, The Netherlands

## Abstract

In three Viewpoints, Sasha Shepperd and colleagues, Geoff Wong, and Aziz Sheikh explore various approaches to help systematic reviewers who wish to review complex health interventions.

## Background to the debate

The UK Medical Research Council defines complex interventions as those comprising “a number of separate elements which seem essential to the proper functioning of the interventions although the ‘active ingredient’ of the intervention that is effective is difficult to specify.” A typical example is specialist care on a stroke unit, which involves a wide range of health professionals delivering a variety of treatments. Michelle Campbell and colleagues have argued that there are “specific difficulties in defining, developing, documenting, and reproducing complex interventions that are subject to more variation than a drug” [10]. These difficulties are one of the reasons why it is challenging for researchers to systematically review complex interventions and synthesize data from separate studies. This *PLoS Medicine* Debate considers the challenges facing systematic reviewers and suggests several ways of addressing them.

## Viewpoint of Sasha Shepperd, Simon Lewin, Sharon Straus, Mike Clark, Martin Eccles, and Ray Fitzpatrick: We Must Address the Conceptual and Analytical Challenges in Synthesising Studies of Complex Interventions

### Background

Over the last two decades, the application of randomised controlled trials and systematic reviews has extended to the evaluation of ever more complex interventions. A range of facets of the complexity of these interventions has been identified. While most health care interventions have some degree of complexity, interventions that include a number of components, which may be independent or inter-dependent, are at the more complex end of the spectrum [1]. Examples include case management and discharge planning, both of which aim to minimise the fragmentation of health care [2],[3]. More recently, complex interventions of a more conceptual nature have been systematically reviewed, such as continuity of care or “trust” between doctors and patients [4],[5]. These present the additional challenge of defining concepts that are often poorly developed.

Compared with single evaluations of complex interventions [1],[6], less attention has been paid to the methodological issues arising from the synthesis of data from complex interventions. Important limitations include difficulties in (1) defining the intervention within the review; (2) searching for and locating relevant evidence; (3) standardising the selection of studies for a review; and (4) synthesising data. In this Viewpoint, we describe the implications of these limitations and suggest some approaches to help systematic reviewers reflect on the conceptual and analytical challenges posed by these types of review.

### 1. Defining the Complex Intervention

To varying degrees, complex interventions can be standardised and defined in individual prospective studies. However, the lack of an agreed definition of complex interventions that have the same aim but are described differently, or inadequately, across studies poses inherent difficulties for systematic reviewers and users of these reviews [7]. Case management illustrates this well. Despite there being no agreed typology, case management has become a generic concept across different specialities to improve inter-professional collaboration and co-ordination of care for individual patients. Case management comes in many forms, including a brokerage model, an integrated care pathway, a liaison service, and self-managed care [2]. All these variations can occur alone or in combination.

Defining the intervention is made difficult when the core purpose of an intervention, such as behavioural counselling [8], varies according to the characteristics of the participants or the trial setting, or if the intervention aims to promote an abstract concept, such as promoting trust or continuity of care [4],[5]. In the field of service delivery, interventions become complex and difficult to define if, as is often the case, (1) they are delivered across the primary-secondary care interface; (2) they are delivered in new settings; or (3) there is an added behavioural dimension and staff perform new behaviours or current behaviours in a new context [9].

Solutions to improve the description and conceptual understanding of the content of a complex intervention include (1) typologies to guide the classification of interventions and (2) supplementary evidence, such as qualitative or descriptive data [10],[11] (see Table 1).

**Table 1:** Identifying the key components of a complex intervention for a systematic review.

Solutions	Examples of Where These Techniques Have Been Used	Is This Prospective or Retrospective?	Additional Data Required	Comments
1. Typologies of the structural characteristics of a complex intervention	Application of the EPOC taxonomy of professional, organisational, and financial interventions to classify quality improvement strategies designed to improve the care of those with type 2 diabetes [12]. In another example, the interpretation by pre-review consensus identified seven categories of occupational therapy intervention for rheumatoid arthritis [16].	Prospective at the review stage.	No	Few typologies exist as this is an evolving field and existing typologies are relatively untested; misclassification can occur [13]; and the mechanism of action for many interventions remains poorly understood [34]. Employing the expertise of those working in the field provides face validity to the categorisation; this is a method that could be applied relatively easily, but problems could occur if consensus is not reached; the results may not be comprehensive.
2. Trial-related data (trial protocols, supplementary studies)	A survey of trialists contributing to a systematic review of stroke units to identify the key attributes of the intervention [18]. In another example, trialists were contacted for their definition of peer support in a review of peer support telephone calls for improving health [35].	Can be prospective [17],[36] or retrospective [18].	Yes	Few examples of supplementary studies to develop intervention typologies [19]; may be problems in generalising the results of these studies across the review.
3. Policy documents	Policy supporting the adoption of a complex intervention can explain subtle differences in the way interventions have been designed and implemented.	Prospective while drafting the protocol for the review.	Yes	Can be difficult to identify the policy documents that correspond to the development of an intervention, particularly as systematic reviews are of the world literature; interventions may not have been developed in response to a particular policy change.
4. Alternative sources of data (qualitative, descriptive) to guide the categorisation of interventions	A qualitative synthesis guided the categorisation of interventions aimed at promoting healthy eating among children and the exploration of heterogeneity [21].	Carried out in parallel with the systematic review of quantitative data.	Yes	A major limitation is the lack of related data [19]; the process is resource intensive and adds another layer of complexity to an already labour-intensive process.
5. Theoretical basis of an intervention	Interventions designed to implement change in practice were categorised according to their theoretical construct [27].	Prospective at the review stage.	No	Theory has seldom been used explicitly to guide intervention development. There are a large number of theories and the empirical basis for many of the behaviour theories remains limited [34].

#### A. Typologies

Typologies can guide the classification of common elements of interventions into homogeneous groups. The Cochrane Effective Practice and Organisation of Care Review Group (EPOC) has developed a typology for interventions aimed at professional practice, organisational, financial, and regulatory systems (see http://www.epoc.cochrane.org/). The Cochrane Consumers and Communication Review Group has developed a typology for consumers' interactions with health care professionals, services, and researchers (see http://www.latrobe.edu.au/chcp/assets/downloads/TopicList.pdf). As an example of how such typologies are used, the EPOC typology was used to classify quality improvement strategies designed for the care of people with type 2 diabetes [12]. Subsequent correspondence highlighted the risk of misclassifying interventions due to inadequate detail [13]. In another example, a typology for heart failure disease management guided the grouping of clinical service interventions (multi-disciplinary, case management, and clinic models) [14],[15].

An alternative is to develop a typology of interventions by consensus. For example, in a systematic review of occupational therapy interventions for rheumatoid arthritis, four occupational therapists identified seven types of intervention (comprehensive therapy, training of motor function, skills training, joint protection, advice on assistive devices, counselling, and provision of splints) [16].

#### B. Supplementary evidence: (i) Trial-related data

Contacting trialists to obtain the protocols they followed, and identifying supplementary research related to the trial, may help define interventions. A qualitative study, conducted alongside a trial of intensive case management for people with severe mental illness, investigated the active ingredients of the intervention in terms of staff roles and organisational features [17]. Team management, comprehensive assessment, and needs-led service were regarded as the key mechanisms of this intervention. In another example, trialists contributing to a systematic review of stroke units were surveyed to build a description of the active components of stroke unit care. These included comprehensive assessment, active physiological management, early mobilisation, skilled nursing care, early rehabilitation, and discharge planning involving carers [18].

#### B. Supplementary evidence: (ii) Qualitative data, descriptive data, and policy documents

When trial-related evidence is inadequate [19], other sources of information may be relevant [20]. A systematic review of barriers and facilitators to healthy eating among children used qualitative evidence, unrelated to the trial data, to gain a better understanding of children's perspectives [21]. The qualitative synthesis guided the categorisation of interventions, according to the degree to which they combined health advice with the promotion of eating fruit and vegetables. A synthesis of qualitative studies aided a fuller understanding of the interventions included in a systematic review of directly observed therapy (DOT) for tuberculosis, by identifying factors that improved adherence [22]. Factors included flexible delivery systems, involving patients in decisions, and social and family support systems [23]. Policy documents can be particularly informative in understanding the development of service interventions across settings, for example, interventions designed to reduce reliance on hospital beds and interventions involving school feeding programmes [24],[25].

#### B. Supplementary evidence: (iii) Theory

Theory may help to explain how an intervention is related to similar interventions in a particular field [26]. However, many reviews fail to locate interventions within a theoretical model. Realist synthesis, which attempts to provide an explanatory analysis of how and why complex social interventions work (or not) in particular contexts, can aid this process. For example, a realist review of school feeding programmes identified the theory and processes that promoted the success of these interventions [25]. Theory can also guide the classification of interventions; behavioural and contingency theory successfully guided the classification of interventions designed to implement change in practice [27]. The theory of planned behaviour was used to address an individual's motivation, attitude, and perceived behavioural control; whereas contingency theory was used to take into account the fit between clinical practice and environmental constraints. However, theory can only improve our understanding of how an intervention works if it is part of an integrated body of knowledge that differentiates the explanatory role of one theory from another and provides robust predictions of causal pathways. An attempt to categorise studies in a Cochrane review of tobacco cessation for young people failed due to the complexity of the interventions and the simultaneous use of several psychosocial theories [28].

### 2. Searching For and Identifying All Relevant Data

The lack of consistent terminology and the inconsistent use of existing terminology to describe complex interventions means that identifying potentially eligible studies can be difficult. Search strategies may be incomplete and risk introducing bias if they identify only a proportion of all possible configurations of a complex intervention. For example, “continuity of care”, a concept that is considered to contribute to high-quality care, can be delivered through numerous mechanisms (shared care, telephone follow-up, patient-held records, and case management, to name a few) [29].

Solutions include characterising the elements of an intervention through an iterative scoping exercise and searching outside the traditional health care domains to include engineering, social sciences, and management journals. Data may be unpublished and only accessed through policy documents, conference proceedings, or book chapters, but need to be obtained to minimise the effects of publication bias [30],[31]. Contacting those working in the field, retrieving references of references, and tracking citations will also increase the efficiency of finding relevant evidence.

### 3. Selecting Studies for Inclusion in a Review

A major threat to validity from an imprecise definition of an intervention is the non-standardised and potentially non-reproducible selection of studies for inclusion in a review. Based on the available information, considerable judgement may be required when assessing how similar any given intervention is to the intervention of interest, particularly for multi-faceted interventions and those at the boundary of the content area. For example, reaching a common understanding of patient-centred interventions was not easily achieved in a review of interventions intended to promote patient-centred care [32].

Solutions to this problem of definition include: (1) refining the definition of an intervention through an iterative process to accommodate previously unseen configurations; (2) contacting study authors for further information; (3) recording the components of an intervention during data extraction; and (4) being explicit in the review about where disagreement occurred.

### 4. Synthesis of Data

Complex interventions with a large number of ill-defined elements may result in a high degree of heterogeneity. Conversely, applying a narrow definition limits generalisability by losing the potential relevance gained from examining an intervention being implemented across a range of settings. A meta-analysis of DOT for tuberculosis provided in clinics, by lay health workers, or in the home provides an example of the usefulness of exploring sources of heterogeneity [22]. The authors found no important difference between DOT and self-administered treatment (risk ratio 1.02, 95% confidence interval 0.86 to 1.21; *I^2^* 64%). However, when the trials were grouped by the location of DOT there was a small beneficial effect for delivering DOT in a home setting compared with self administration (risk ratio 1.10, 95% confidence interval 1.02 to 1.18; *I^2^* 53%). This beneficial effect allows for several possible interpretations; for example, the burden upon patients of travelling to a clinic five days a week is minimised by having their therapy supervised at home by a lay health worker or a community or family member.

Solutions to the problem of data synthesis include categorising interventions by key variables and retaining these in the analysis. For example, a meta-analysis of discharge planning and post-discharge support categorised interventions by intensity, which varied from a single home visit, to increased clinic follow-up with telephone contact through to extended home care services [3]. If meta-analyses cannot be performed, similar processes can be conducted whilst performing narrative synthesis. The quality of narrative analysis and applicability of review findings have recently received more attention [33] (see Box 1).

**Box 1. Presentation of Review Findings: Information To Support Assessment of the Applicability of Evidence of Effectiveness**

**Intervention Content** [**1**]

- Describe the content (the active ingredients) of the interventions
- Describe any interventions received by the control group, including the content of “usual care”
- Describe how the interventions were delivered and any differences in delivery across the included trials
- Describe the contextual similarities and differences between the trials

**Intervention Fidelity**

- Include details describing whether the interventions included in a review do what is intended or if they deviate from the intended shape or form during the course of implementation
- Include an assessment of whether an intervention failed because it was poorly implemented or it was not effective

**Intervention Sustainability**

- Include details on the sustainability of intervention effects over time

**Roll Out/Scaling Up of the Intervention** [**7**]

- Report data on accessibility, the risk of adverse events, cost-effectiveness, or budget impact of interventions
- Address the following questions regarding the applicability of the evidence to individual patients (where applicable) [37]:
  - Have biological results (e.g., sex, co-morbidities, age) that might modify the treatment response been excluded?
  - Can consumers comply with the treatment requirements?
  - Can health care providers comply with the treatment requirements?
  - Are the likely benefits worth the potential risks and costs?
- Address the following questions regarding the applicability of the evidence in other health systems (where applicable) [38]:
  - Are there important differences or similarities in the structural elements of health systems or of health services between where the research was done and where it will be applied that might mean that an intervention could not work in the same way?
  - Are there important differences in on-the-ground realities and constraints (i.e., governance, financial, and delivery arrangements) between where the research was done and where it could be applied that might substantially alter the potential benefits of the intervention?
  - Are there likely to be important differences in the baseline conditions between where the research was done and other settings? If so, would this mean that the intervention could have different absolute effects, even if the relative effectiveness was the same?
  - Are there important differences in perspectives and influences of health system stakeholders between where the research was done and where it could be applied that might mean an intervention will not be accepted or taken up in the same way?

### Conclusion

Despite the range of supplementary methods available to improve the synthesis of complex interventions, most of these methods are infrequently used. There are several reasons for this lack of use. In some cases the theory underpinning a specific complex intervention has not been assembled. However, there are usually few data reporting the characteristics of complex interventions, and what data there are tend to be of poor quality. Although simple in concept, providing an adequate description of complex interventions can be technically difficult. The need to address this is becoming urgent as interventions with multiple components evolve in response to the complex health problems faced by health services. Current criteria to improve the reporting of health research are primarily concerned with the internal validity of studies [6], and while these include criteria related to the intervention, these guidelines can still be followed without providing adequate details of the intervention (see Table 2). It is essential that methods to improve the descriptions of complex interventions are further developed and tested with the expectation that they will complement existing systematic review methodology.

**Table 2:** Current guidance for reporting complex interventions and where further research is required.

Reporting Tool [6]	Guidance for Reporting the Characteristics of an Intervention	Gaps	Where Further Research Is Needed To Improve the Reporting and Synthesis of the Effects of Complex Interventions
CONSORT for non-pharmacologic treatments such as surgery, technical interventions, rehabilitation, psychotherapy, behavioural interventions, implantable and non-implantable devices, and complementary medicine *(extension of the CONSORT statement for reporting randomised controlled trials)*	Details of the intervention and the experience of the care provider for therapist-dependent interventions	Does not address fidelity or external validity; can be applied in a non-systematic fashion	**Individual Studies**• Systematise the description of complex interventions in trial reports as well as in the reports of studies using other evaluation designs• Ensure that more attention is given in reporting to issues of intervention fidelity and external validity, such as the characteristics of interventions, the health care system, and the setting and implementation• Increase the use of qualitative and quantitative process evaluations alongside trials• Ensure that the development of interventions for new trials is better informed by the findings of existing systematic reviews and by existing typologies and frameworks
QUOROM *(Quality of Reports of Meta-Analysis of Randomised Controlled Trials)*	Details of the intervention	Does not provide detailed guidance	
Transparent Reporting of Evaluations with Non-Randomized Designs (TREND) [39] *(developed for reporting behavioural and public health interventions)*	Details of how and when interventions were delivered, the content, the delivery, location, and setting, number of sessions, time span, and external validity	Provides the most detailed guidance, to be empirically tested	**Research Synthesis**• Formalise methods to identify and disaggregate the key elements and effects of a complex intervention within a systematic review to complement existing systematic review methodology• Refine methods to identify relevant qualitative data linked to trials• Systematise the description of complex interventions in systematic reviews• Develop empirically based typologies of interventions, linked to their hypothesised mechanisms of actions and effect, and test how well these work

## Geoff Wong's Viewpoint: We Must View Complex Health Interventions in a New Way

I believe that complex health interventions (CHIs) *can* be systematically reviewed, but only if a paradigm shift occurs in the way that these interventions are conceptualised. In this Viewpoint, I discuss an alternative way of viewing CHIs that focuses on the interactions between components of a CHI and the impact of human behaviour on the outcome of the intervention. I then discuss how this different way of viewing CHIs has given rise to a different method, called “realist review”, to systematically review CHIs.

### CHIs Are Non-Linear and Produce “Irregular” Outcomes

CHIs are more than just a complicated “jumble” of components that interact in a regularly predictable but linear fashion (i.e., deterministically) to produce health outcomes. Interactions between the components are not as deterministic as might be expected because CHIs are highly dependent on human behaviour [1],[40]. The “components” in CHIs are invariably made up of people (e.g., researchers) trying to get other people (e.g., study participants) to “do” or “not do” something (e.g., to stop smoking in a CHI of smoking cessation). The actions taken by the “human components” of CHIs are influenced by the context in which the intervention takes place. Taking the example of smoking cessation, the actions that smokers can take will depend on their personal circumstances, including their health status, while the actions the clinic staff can take will be guided by factors such as the trial protocol and the clinical setting.

Thus the context in which CHIs take place is a key variable, because context influences and limits the range of people's choices and actions. Such context explains why the interactions between the components of CHIs are not deterministic and why the outcomes of CHIs can vary when the CHI is repeated. There will be a myriad of different contexts, each having a slightly different influence on those involved in the intervention. Human behaviour varies under the influence of different contexts—and so the pattern of health outcomes achieved from CHIs are best described as “demi-regular”.

Shepperd and colleagues rightly point out that the systematic review of CHIs remains challenging. From the range of solutions they have proposed, the greatest progress is likely to be made by focusing on theories (Box 2) that can explain and “predict” how certain contexts influence individuals to act in certain ways to produce certain outcomes. Pawson and colleagues have already made some progress towards such explanation and “prediction” using the “realist review” method of systematic review [41].

**Box 2: Definitions of Theory**

**Theory:**

There are multiple definitions for theory, and in this Viewpoint article: “A theory is an attempt to organize the facts—some ‘proven’, some more conjectural—within a domain of inquiry into a structurally coherent system” [45].

**Middle-Range Theory:**

This is a theory that lies “…between the minor but necessary working hypotheses that evolve in abundance during day-to-day research and the all-inclusive systematic efforts to develop a unified theory that will explain all the observed uniformities of social behavior, social organization and social change…

“It is intermediate to general theories of social systems which are too remote from particular classes of social behavior, organization and change to account for what is observed and to those detailed orderly descriptions of particulars that are not generalized at all. Middle-range theory involves abstractions, of course, but they are close enough to observed data to be incorporated in propositions that permit empirical testing.” [42].

### Realist Review

Realist review is a systematic review method that focuses more on trying to explain as opposed to judge CHIs. As such, it seeks not so much to answer the question of “If” a CHI works, but “How”, “Why”, “In what circumstances”, “For whom”, and “To what extent” it works. The underlying premise of realist review is that the demi-regular patterns of interactions between the components (so-called “demi-regularities”) that make up CHIs with similar goals can be explained by middle-range theory (Box 2) [42]. For any similar group of CHIs (e.g., smoking cessation interventions), the myriad of contexts influencing behaviour so as to generate outcomes are not impediments to realist review, but act as the “raw materials” from which demi-regularities can be identified. Middle-range theory (or theories) are then sought to explain why these demi-regularities occur. As the review progresses iteratively, theories that “work” (i.e., best explain sets of demi-regularities) are repeatedly “tested” against the observations reported in each CHI included in the realist review [41],[43],[44].

In exploring the feasibility of systematically reviewing studies that evaluate CHIs, I believe we have come to a crossroads. Currently, the dominant systematic review methodology and paradigm is based on the Cochrane review. Without doubt, its dominance has been well earned as its usefulness in the systematic review and meta-analysis of pharmaceutical and other “simpler” medical interventions has advanced the evidence-based practice of medicine. However, efficacy studies of pharmaceutical studies are less reliant on the human agency that we see in CHIs. In other words, a well-characterised “drug” does things to people to cause outcomes in a deterministic way, and context is much less relevant. Once human agency comes into play (as can be seen in the differences in outcomes reported between pharmaceutical efficacy and effectiveness trials), then context starts to play a bigger role. By the time we come to CHIs (where outcomes are highly dependent on human agency), then the appropriateness of the current dominant systematic review method needs to be questioned.

### Conclusion

In CHIs, the action of individuals under specific contexts results in outcomes. The way these outcomes result is neither deterministic nor regular, but can be explained and “predicted” by middle-range theory. Systematically reviewing CHIs is only feasible when the review method takes into account these properties, and theory-driven reviews are our best bet.

## Aziz Sheikh's Viewpoint: Undertaking Meaningful Systematic Reviews of Complex Interventions Is Inherently Complex

The evaluation of complex interventions is increasingly commonplace within health services research. It is therefore important and timely that attention is given to reflecting critically on how these studies can best be identified and appraised, and their findings then synthesised and interpreted. The answer to the somewhat rhetorical question of whether it is feasible to systematically review studies of complex interventions is, at one level at least, obvious. Many of the general principles of systematic review methods can and indeed should be used when undertaking systematic reviews of complex intervention studies in health care. Sasha Shepperd and colleagues offer a number of useful suggestions in this respect, including: (1) the need to use broad search techniques in an attempt to identify and include studies that may have been poorly indexed; (2) carefully defining and describing the interventions being studied; and (3) a willingness to consider narrative synthesis if studies are found to be too heterogeneous to be synthesised quantitatively.

There are, however, a number of important conceptual and practical challenges in undertaking such reviews. One key consideration that is usefully highlighted by Geoff Wong is the importance of studying contextual considerations, both in relation to understanding the broader picture in which individual studies have been conducted, and in order to make sense of how the landscape has (often irrevocably) changed as a result of the intervention being applied. Wong also helpfully discusses the central importance of considering not only *what* was achieved in relation to standard parameters of interest, such as effect size, but also *why* this may have been achieved, i.e., a clear description of the processes that the intervention has operated through in order to achieve this effect [46]. This latter consideration is particularly important in that it is these processes that are far more likely to prove generalisable than the specifics of the intervention under study [47].

Based on my experiences of conducting a number of complex intervention trials and attempts at undertaking systematic reviews of complex interventions, I am increasingly of the opinion that there are a number of additional unresolved issues that warrant more detailed reflection.

Considering first the definition and description of the intervention, it is important that researchers not only describe the intervention in detail, but also that they capture and describe how the intervention may have evolved during the course of delivering it. A somewhat extreme example of such a complex health care intervention, but which should nonetheless illustrate the point well, is the United Kingdom's National Programme for Information Technology. This programme is the largest non-military information technology-based intervention in the world. Over the course of its relatively short life course, the programme has undergone re-branding, had several rounds of leadership change and, more fundamentally, expanded its list of core deliverables [48]. Such changes are often mirrored, albeit on a smaller scale, in the health interventions that we more routinely consider as complex interventions. The key issue here is that such modifications should not be seen as compromising the fidelity of the intervention. Instead, these modifications should be recorded and described as fully as possible in order to allow readers to make sense of what modifications were considered necessary and why, and through so doing allowing readers to appreciate what was *actually* delivered [1].

A second important consideration relates to what is and what is not considered a complex intervention. Most interventions do have a degree of complexity—even a “simple” aspirin effectiveness trial has some complexity—because the processes through which the delivery of the intervention leads to actual patient compliance with the treatment is inevitably made up of “several interacting components” [1]. Some argue that this in effect means that the term “complex intervention” is somewhat meaningless. I would take a somewhat different view, stressing that what we must *not* do is take the other extreme of excluding from our frame of reference interventions that are judged so complex that they are not amenable to study through randomised controlled trial designs. Many health policy initiatives, particularly those that are national or supra-national, are inherently multi-faceted, such as national legislation prohibiting smoking in public places and the National Programme for Information Technology. Such programme-level interventions are often not easily amenable to study using quasi-experimental designs, but their evaluation remains important and such interventions should also be studied through systematic reviews.

My third point relates to the importance of elucidating the likely mechanisms through which the effects of the intervention are mediated [49]. Carefully theorised studies/interventions can help greatly in this respect, as can accompanying embedded qualitative work, particularly if it has a longitudinal dimension [50],[51]. One way forward is to assign greater weight to studies that have described mechanisms, but a more general point is the importance of searching for and including relevant theoretical and qualitative work into complex intervention systematic reviews.

Considering then the question of meta-analysis, whilst this may be appropriate in some systematic reviews of complex interventions [52], meta-analysis may also often be somewhat inappropriate. Instead, we should really be more interested in understanding the ways in which local contextual considerations may have acted as co-factors in helping shape delivery of the intervention, rather than focusing on finding any overall summary effect of the intervention. Such summary effects may tend to obscure rather than enlighten [25]. Consider, for example, our phase III and IV studies of the accessibility, acceptability, and effectiveness of a telephone-based review service for hard-to-reach people with asthma [53],[54]. These studies could only have been possible in a climate that has a well-developed information technology infrastructure, including routine use of electronic health records, values regular review of those with long-term conditions, and rewards practitioners accordingly. Paying careful attention to such crucial contextual influences allows readers to begin to assess the role of co-factors in shaping delivery of the intervention and also in assessing its likely generalisability.

The upshot of all of this is that systematic reviews of complex health interventions can and should be done, but if they are to shed more light than darkness, the systematic reviewers need explicitly to consider doing two things. First, they should search for and include relevant theoretical and qualitative work. Second, where relevant, they should include data from a broader range of experimental study designs than is currently normally the case in most Cochrane systematic reviews. Such an approach will in turn necessitate development of better search strategies to locate this non-trial literature and also the availability of techniques for the quality assessment of such studies. Theory-driven analysis, wherever possible, should also accompany the more conventional quantitative syntheses, the emphasis on the latter being down-played. If the Cochrane Collaboration can take a lead in spearheading and supporting these developments, this would represent a considerable service to this important and expanding field of evidence synthesis.

## Abbreviations

CHI: complex health intervention
DOT: directly observed therapy
EPOC: Cochrane Effective Practice and Organisation of Care Review Group
